# Conformational Control of DNA Origami by DNA Oligomers, Intercalators and UV Light

**DOI:** 10.3390/mps4020038

**Published:** 2021-05-22

**Authors:** Ruixin Li, Haorong Chen, Hyeongwoon Lee, Jong Hyun Choi

**Affiliations:** School of Mechanical Engineering, Purdue University, West Lafayette, IN 47907, USA; li2594@purdue.edu (R.L.); haorong.c@gmail.com (H.C.); lee3807@purdue.edu (H.L.)

**Keywords:** DNA origami, conformation, stress, strand displacement, intercalator, UV

## Abstract

DNA origami has garnered great attention due to its excellent programmability and precision. It offers a powerful means to create complex nanostructures which may not be possible by other methods. The macromolecular structures may be used as static templates for arranging proteins and other molecules. They are also capable of undergoing structural transformation in response to external signals, which may be exploited for sensing and actuation at the nanoscale. Such on-demand reconfigurations are executed mostly by DNA oligomers through base-pairing and/or strand displacement, demonstrating drastic shape changes between two different states, for example, open and close. Recent studies have developed new mechanisms to modulate the origami conformation in a controllable, progressive manner. Here we present several methods for conformational control of DNA origami nanostructures including chemical adducts and UV light as well as widely applied DNA oligomers. The detailed methods should be useful for beginners in the field of DNA nanotechnology.

## 1. Introduction

Besides its essential biological functions, deoxyribonucleic acid or DNA can also serve as information-rich materials that self-assemble into custom-designed structures with nanometer precision [1,2,3]. The rational use of DNA molecules has emerged as the field of structural DNA nanotechnology [4,5,6]. The DNA origami, a type of DNA nanostructures formed by using multiple short single-stranded DNA (ssDNA) oligomers (termed staples) to direct the folding of a long ssDNA (termed scaffold) into a designed pattern, has garnered much attention due to its programmability and complexity [7,8]. DNA origami may form complex one-dimensional (1-D), 2-D and 3-D nanostructures of arbitrary geometries, including smiley faces [7,9], multi-tooth gears [10], nanorobots [11,12], polyhedral [13], and auxetic structures [14,15]. These nanostructures have been explored as static templates for organizing proteins [16,17], small molecules [18,19], and nanoparticles [20,21,22,23,24]. It is also possible to use them as dynamic platforms for sensing and actuation at the nanoscale by changing their shapes in response to environmental cues or user demands [25,26]. To enable such reconfigurations, a structure needs to have moving or deformable parts, and a source of energy is required to drive the structural transformation process.

The most widely explored shape-changing mechanisms are based on ssDNA hinges connecting two or more rigid parts. With the flexibility of single strands, the connected rigid parts can move freely about the hinge, unless constrained by further binding interactions, which drives the reconfiguration. It can be generally understood as a “close/open” process; the structure closes when linking ligands (e.g., oligonucleotide DNA) bind the moving parts together, and the structure opens when the ligands are disengaged often via toehold-mediated strand displacement [27,28,29,30,31]. Similar methods have been summarized in recent reviews [32,33,34].

**Figure 1:**
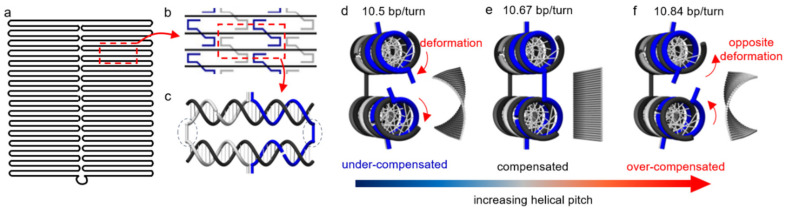
Schematics of the conformational change in a DNA origami. (**a**) Layout of scaffold pattern in a rectangular origami tile. (**b**) A section of the origami tile indicating the periodic arrangement of the staples (blue and gray) against the scaffold (black). (**c**) The repeating segment indicated as a red dashed box in (**b**). The staples (blue and gray) pair with the scaffold (black), forming double helices connected by crossovers (indicated by blue dotted circles). There are 32 bp between the adjacent crossovers for three turns, corresponding to 10.67 bp/turn. (**d**–**f**) Side views of the periodic arrangement in (c) along with corresponding origami conformations from finite element simulations using CanDo on the right. (**d**) The DNA duplexes experience significant torsional deformation under natural conditions (10.5 bp/turn) in order to maintain crossover connections. The helical mismatch results in a right-handed twist over the entire origami tile. If the mismatch is compensated, crossovers will form without distortion. As such, the tile will be perfectly flat (**e**). Over-compensating the mismatch (e.g., 10.84 bp/turn) will lead to a left-handed twist in the origami tile (**f**). (Contents in this figure were previously published. Adapted with permission from [42]. Copyright (2016) American Chemical Society. Further permissions related to the material excerpted should be directed to the ACS).

There are other, less explored mechanisms for reconfiguration, which are based on the deformation of double-stranded regions. These regions are often treated as rigid but actually possess considerable deformability. For a Watson-Crick B-form DNA duplex, the intrinsic helical pitch is about 10.5 base-pairs (bp) per turn [35,36]. The pitch, however, may not match exactly with the origami design, which can cause a mismatch, and thus, internal stresses. For example, Figure 1 illustrates such helical mismatch in the origami design where arranging 32 bp between two neighboring crossovers (Figure 1b,c) for three turns or 10.67 bp/turn may induce right-handed torque and twist [37,38]. In contrast, a helical pitch greater than the designed 10.67 bp/turn, if possible, will result in left-handed torque and deformation. Since the double helices behave similar to springs whose geometries change continuously in response to applied stresses, a modulation of the stresses could lead to the control of origami conformation in a progressive manner [39,40,41].

**Figure 2:**
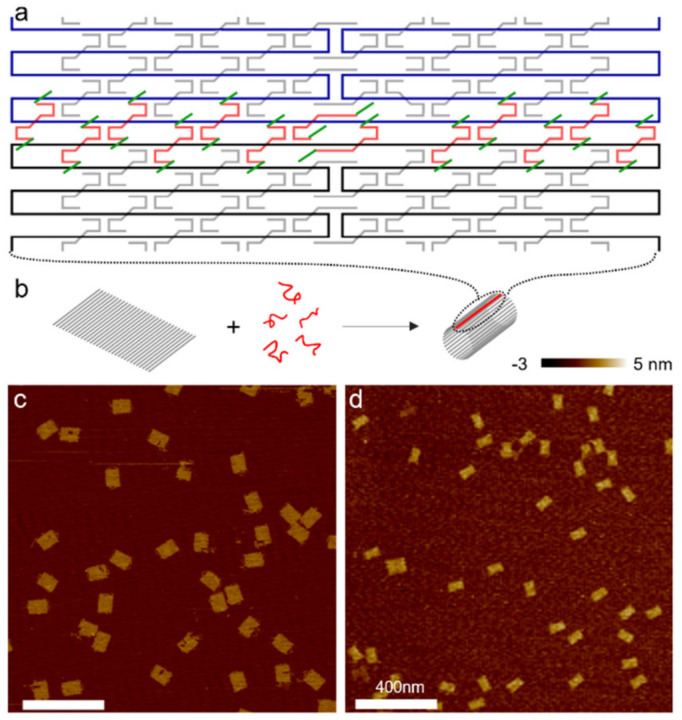
(**a**) Schematic of a portion of a single-layer DNA origami rectangle with its upper and lower boundaries (black and blue) connected and sealed by a set of linker strands (red) with toeholds (green) at both ends. Staple strands in the origami tile are shown in gray. (**b**) Schematic of DNA origami cyclization. A flat tile bends and cyclizes into a short cylinder with the help of the linkers (red). Gray rods represent DNA double helices. The sealed boundaries are indicated by red lines. (**c**,**d**) AFM images of the two origami tile species. (**c**) All origami tiles formed by thermal annealing are flat. (**d**) Cyclized DNA origami after incubation of flat tiles with linkers at 50 °C for 2 h. The majority of DNA origami measures half the length of the flat tile with twice thickness. (Contents in this figure were previously published. Adapted with permission from [37]. Copyright (2014) American Chemical Society).

Here we present a series of conformational control approaches that span both categories. The control strategies include DNA-intercalators and UV light as well as DNA oligomers. Utilizing DNA oligomers as control signals is demonstrated with DNA linkers and releasers. In this protocol, this scheme is discussed using three examples: (1) hierarchical assembly of DNA origami cylinders into an elongated hollow tube, (2) reversible cyclization of a rectangular DNA origami tile, and (3) structural modulation of DNA origami nanocages for guiding liposomes. In the first example, a set of linkers serve as the ligands to bind two free-moving origami cylinders together, while the releasers are designed for disengaging the linkers from the cylinders, thereby separating them [43]. To enable the function of releasers, an 8-nucleotide (nt) long toehold with distinct sequences is added to both ends of every linker strand. During the disengaging process, the releasers bind to the toeholds first, and undergo the strand displacement due to a complete complementarity [28]. Short DNA origami cylinders are synthesized and linked into an elongated tube, which can then be disassembled into separate cylinders by corresponding releasers. The disassembled cylinders may be subsequently linked into a tube of a different chirality from the previous tube using another set of linkers [43]. This reconfiguration process via hierarchical assembly may be repeated multiple times. In the second example (Figure 2), a set of DNA linkers is used to connect two opposite edges of an origami tile, forming a short cylindrical structure [37]. The cyclization of a single-layer origami rectangle can be efficiently reversed via toehold-mediated strand displacement using releaser strands. This process depends strongly on the temperature and structural geometry. In the third case, cylindrical units with four pillars are assembled into long tubular cages by a set of DNA linkers [44]. They can be disengaged by releasers, which is similar to the first example. In addition, the pillars in each unit may be removed by adding displacement oligos (similar to releasers). If all four pillars in each unit are removed, the tubular cages will be shorter. This reconfiguration would merge all the small liposomes immobilized by each unit together. If only two neighboring pillars in each unit are disassembled, the tubular cages will bend to one side and assume a partial torus shape. Such a transformation bends the large liposome inside the cage.

**Figure 3:**
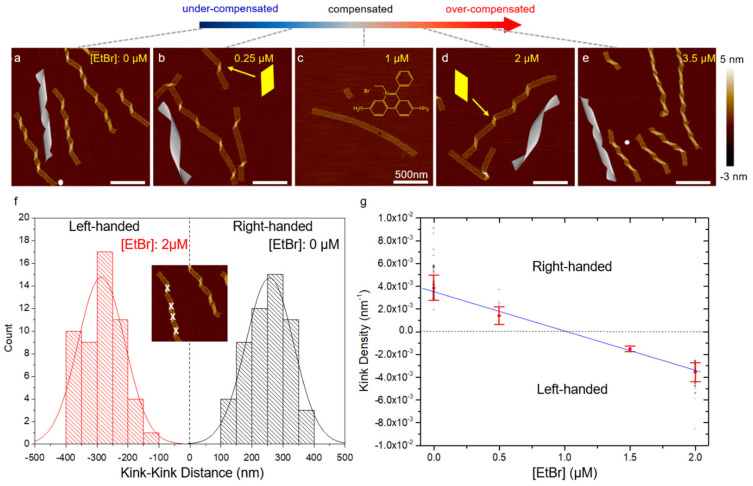
(**a**–**e**) AFM images of DNA origami ribbons (2 nM) as a function of EtBr concentration from 0 to 3.5 µM. CanDo simulation results for 10.5 to 10.8 bp/turn are also shown. The handedness of the ribbon twist is represented by the shape of the parallelogram kinks highlighted in (**b**,**d**). The added EtBr intercalates into DNA duplexes, unwinding the DNA helicity and increasing the helical pitch. As a result, the ribbon conformation gradually changes from a right-handed twist to flat and then to a left-handed curvature. The progressive conformational change is characterized by the kink density. (**f**,**g**) Statistics of kink density in the ribbons. (**f**) Histogram of kink-kink distance measured under two EtBr concentrations: 0 and 2 µM. In the inset, the parallelogram-shaped kinks indicating the degree of origami twisting are marked using software ImageJ. Distances between the right-handed and left-handed kinks are marked as positive and negative, respectively. The statistics follow Gaussian functions in general. (**g**) Kink density, defined as the inverse of the distance between neighboring kinks, decreases as EtBr amount increases. The plot suggests that flattening is expected to occur at approximately 1 µM EtBr, which agrees well with experiment. The inverse of the average distance and the associated standard deviation are denoted by red symbols. The black symbols indicate the scattering of the measurements. The blue line suggests the progressive control of origami conformation using intercalators. (Contents in this figure were previously published. Adapted with permission from [42]. Copyright (2016) American Chemical Society. Further permissions related to the material excerpted should be directed to the ACS).

Intercalators and UV light are also introduced as powerful mechanisms for modulating conformation of a ribbon from polymerization of rectangular DNA origami tiles by controlling the internal stress of the structure. In this approach, the origami tile is designed with a helical pitch of 10.67 bp/turn, which is slightly larger than 10.5 bp/turn (Figure 3a). This difference creates internal stresses and results in structural deformation as discussed above. In Figure 3a, the stress-induced distortion is manifested by parallelogram-shaped kinks. Modulation of the helical pitch should thus control the origami conformation and the degree of deformation.^42^ Intercalators such as ethidium bromide (EtBr) are inserted between base-pair stacks which unwind the DNA double helix [45,46], and thereby, modulate the helicity mismatch. This simple strategy has been proven to effectively change the internal stress of DNA origami [47] and global conformation [48]. When the intercalator concentration increases gradually, the helical pitch will vary from inherent 10.5 bp/turn to designed 10.67 bp/turn and even greater, for example, 10.84 bp/turn. Accordingly, the tile should experience a progressive structural modulation from an original right-handed twist to flat, and to even left-handed deformation (Figure 3).

Beside EtBr, there are a number of DNA intercalators, including YOYO-1, doxorubicin, TOTO-3, etc., [49,50,51,52,53]. Similarly, they can alter the DNA helicity by inserting into the base-pair stacks. Some intercalators may behave differently from EtBr. For example, doxorubicin can also bind with minor groove at A/T rich domains in addition to intercalation. Their functionality is almost the same, nonetheless. For a comprehensive understanding and diverse options, we discuss below the protocols related to SYBR Green I [54], BMEPC [55], and daunorubicin [56].

**Figure 4:**
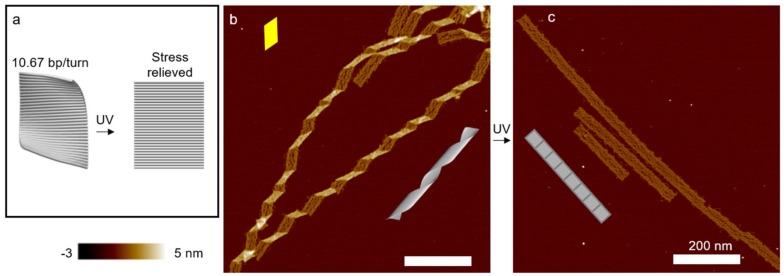
Effect of UV light on DNA structures. (**a**) Schematic of an origami rectangle. The tile is designed at 10.67 bp/turn, and the internal stress causes a global curvature. Moderate UVC or UVB radiation flattens the rectangular origami tile. (**b**) AFM image of polymerized DNA origami ribbons. The curvature of the individual tiles leads to a heavy twist of the ribbons. The parallelogram-shaped kinks indicate the right-handed twist (also see Figure 3). (**c**) AFM image of DNA ribbons after UVC irradiation (~2.5 kJ/m^2^). The origami structures are completely flat. (Contents in this figure were previously published. Adapted with permission from [60]. Copyright (2017) American Chemical Society. Further permissions related to the material excerpted should be directed to the ACS).

UV light can also affect the DNA structures and the effect strongly depends on the wavelength. Short- and medium-wavelength UV radiation (termed UVC and UVB, respectively) can photochemically create minor lesions such as cyclobutane pyrimidine dimer (CPD) [57,58]. That is, thymine (T) and/or cytosine (C) will be photoexcited and react with adjacent pyrimidine bases, forming dimeric photoproducts. This type of reaction is not limited to two Ts or two Cs. It can also combine a T and a C together. The yields of the three species are different; TT and TC are comparable, while CC is significantly lower [59]. The sequence of the DNA origami tile is determined by the M13mp18 scaffold. The ratio of A/T in the scaffold is about 58% and they are distributed almost randomly. As such, it is impossible to find a region with significantly high TT or TC content. Overall, the UV-induced effects will be nearly uniform across the DNA origami, while they are a function of irradiation dosage. It is also worth mentioning that AFM is unable to depict such small changes after photoreaction. This type of photochemical reactions can disrupt DNA base-pairs and release inherent internal stresses. As a result, the origami tile with a right-handed twist due to a helical pitch of 10.67 bp/turn can experience a global “flattening” effect as shown in Figure 4 [60]. A controlled exposure of UV irradiation can lead to a progressive conformation change.

**Figure 5:**
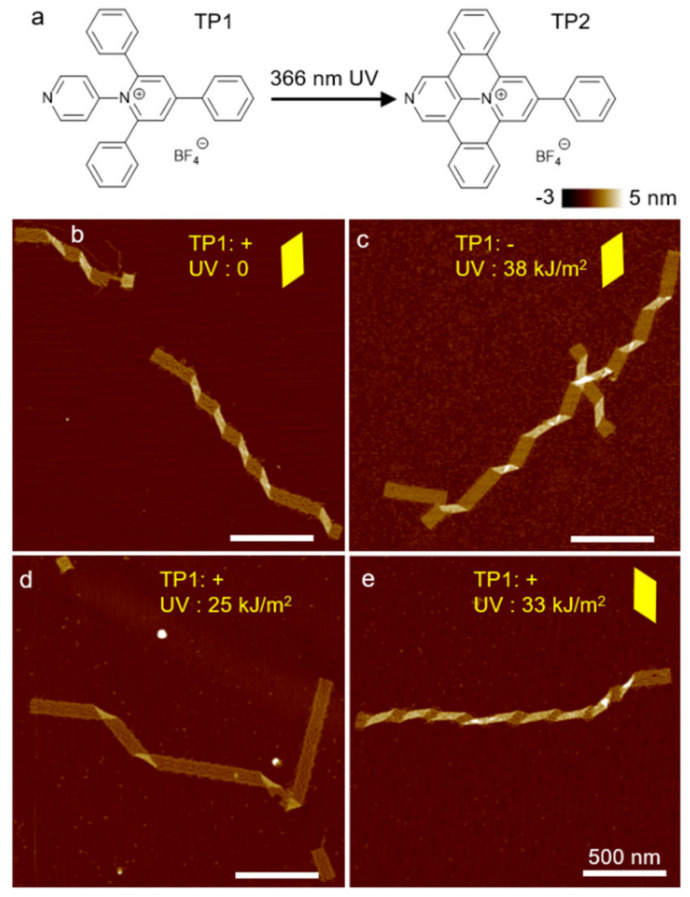
(**a**) Chemical structure of photoswitchable intercalator: TP1 and TP2. TP1 does not associate with DNA. UVA converts TP1 to TP2, which is a strong DNA intercalator. (**b**–**e**) AFM images of origami ribbons with and without TP1 and UVA radiation. (**b**,**c**) If one of the two components is absent, there is no change in the origami structure. (**d**,**e**) Conformation change occurs only when both TP1 and UVA are present simultaneously. (**d**) Partial conversion of TP1 to TP2 flattens DNA structures. (**e**) Complete conversion can change the DNA helicity significantly, causing a flip of twist handedness. Yellow parallelograms highlight the shape of the kinks, suggesting twist-handedness. (Contents in this figure were previously published. Adapted with permission from [60]. Copyright (2017) American Chemical Society. Further permissions related to the material excerpted should be directed to the ACS).

Long-wavelength UV light (termed UVA), on the other hand, shows a minimal impact on DNA, yet it can stimulate photoactive chemical adducts [61], thereby leading to structural modulations without significant side effects. For example, a triarylpyridinium cation (TP1) can be biscyclized into a polycyclic form (TP2) by UVA radiation [62]. TP2 is a strong DNA intercalator, whereas TP1 does not interact with DNA. Therefore, UVA radiation in the presence of TP1 can significantly change the twisting state of the ribbon from polymerization of origami tiles as shown in Figure 5. In contrast, the origami will not be significantly affected by the addition of TP1 or by UVA radiation alone. Overall, a combination of UV radiation and photoactive DNA adducts can gradually change the shape of DNA origami ribbon. Below we present the detailed procedures of conformation control of DNA origami using DNA oligomers, intercalators, and UV light.

## 2. Materials

All samples and buffers are prepared in deionized (DI) water (resistance = 18 MΩ). All chemical compounds are purchased from Sigma Aldrich unless specified otherwise.

### 2.1. DNA Origami

1. All DNA oligomers (including staples, linkers, and releasers) are obtained from Integrated DNA Technologies (75 µM), and are stored at −20 °C.
2. M13mp18 scaffold (7249-nt) is supplied by Bayou Biolabs (0.5 µg/µL), and is stored at −20 °C.
3. TAEM buffer: 40 mM trisaminomethane, 20 mM acetic acid, 1 mM ethylenediaminetetraacetic acid (EDTA) disodium salt, and 12.5 mM magnesium acetate (pH ~8).

### 2.2. DNA Adduct

1. Ethidium bromide (EtBr) is supplied by BIO-RAD (10 mg/mL) in an opaque plastic bottle.
2. 3,6-*bis*[2-(1-methylpyri-dinium)ethynyl]-9-pentyl-carbazole diiodide (BMEPC) is prepared following the procedure published elsewhere [63]. It is stored in dark at 4 °C.
3. Daunorubicin HCl is acquired from Santa Cruz Biotechnology (0.01 M).
4. Triarylpyridinium (TP1) is synthesized according to the procedure described previously [62]. It is stored as saturated aqueous solution in dark.

### 2.3. Imaging Buffer

1. MES buffer: 50 mM 2-(N-morpholino)ethanesulfonic acid (MES), 5 mM magnesium acetate, and 200 mM NaCl (pH ~6.5).
2. Fixing buffer: 40 mM trisaminomethane, 20 mM acetic acid, 1 mM EDTA disodium salt, 12.5 mM magnesium acetate, and 2 mM nickel chloride (pH ~8).

### 2.4. Equipment

1. BIO-RAD S1000 Thermal Cycler: The cycler can bring PCR tubes in its wells to pre-selected temperature(s) for pre-set time. It is used for thermal annealing and incubation of DNA origami.
2. UVP lamp (model UVGL-25): The lamp can emit UV light centered around 254 and 366 nm which may be switched by applying short- and long-wavelength settings, respectively. It is used as the source of UVC and UVA.
3. Spectroline TE-312S UV Transilluminator: The transilluminator can radiate UV light peaked at 312 nm. It severs as the source of UVB.
4. Homemade quartz tube (inner cross-section size: 2 × 4 mm^2^): Unlike a glass tube, the quartz tube is transparent in the UV spectrum, thus suitable for UV irradiation.
5. Bruker Dimension Icon atomic force microscopy (AFM) with SCANASYST-AIR probes: AFM is high-resolution scanning force microscopy, which uses a laser beam to detect the deflection of a cantilever with a tip scanning the sample, normally on a flat surface. For the Bruker AFM equipped with given probes, Peak-Force tapping mode is used for scanning, which can acquire high-quality imaging in air phase without damaging the sample significantly.

## 3. Methods

### 3.1. DNA Origami Assembly

1. Assembly of origami units (e.g., cylinders and rectangular tiles): Find the staple strands corresponding to DNA origami cylinders and rectangular tiles, and group them into two sets (one set for each origami). For synthesis either origami, mix 10 nM scaffold strands with 3.5× staple strands in TAEM buffer (see Note 1, 2 and 11). The final volume is around 55 µL (see Note 3). Thermally anneal the mixture from 75 to 4 °C at −1 °C/minute (see Note 4). After the annealing, store the mixture at 4 °C.
2. Assembly of DNA origami ribbons: Find the linker strands corresponding to polymerization of DNA origami tiles and group into one set. Mix 10 nM DNA origami tiles with 10× linkers and incubate the mixture at 40 °C for 1 h. The total volume is approximately 57 µL (see Note 5). After the incubation, store the mixture at 4 °C.

### 3.2. Reconfiguration of DNA Origami Cylinders Using DNA Oligomers

1. Assembly: Mix 10× linkers with 10 nM DNA origami cylinders. The volume is about 57 µL (see Note 5). Then incubate the mixture at 40 °C for 1 h. After the incubation, store the mixture at 4 °C.
2. Disassembly: Add 20× releasers with elongated tubes assembled from origami cylinders (see Note 6). The volume is approximately 60 µL (see Note 5). Then incubate the mixture at 40 °C for 6 h. After the incubation, store the mixture at 4 °C.
3. Reassembly: Mix 40× new linkers (different from the linker set for initial assembly) with disassembled origami cylinders for a final volume of 67 µL (see Note 5). Then incubate the mixture at 40 °C for 1 h. After the incubation, store the mixture at 4 °C.

### 3.3. Reconfiguration of DNA Cage Units

1. Assembly: Mix 20× linkers with 50 nM DNA origami cylinders. Then, incubate the mixture from 40 °C to 20 °C overnight (see Note 7).
2. Disassembly/reconfiguration: Add 30–60× releasers for disassembly of the cage units or the pillars in each cage unit. Then incubate the mixture at 44 °C overnight (see Note 7).

### 3.4. Cyclization of DNA Origami Tiles by DNA Oligomers

Dilute DNA origami tiles to 1.3 nM with TAEM buffer and then mix with 154× linkers. The total volume is approximately 30 µL. After that, incubate the mixture at 50 °C for 2 h and store the mixture at 4 °C (Figure 2).

### 3.5. DNA Intercalation

1. EtBr: Dilute the DNA origami ribbons to 2 nM (see Note 6) with MES buffer and mix with concentrated EtBr solution to reach different final concentrations from 0 to 3.5 µM of EtBr. The final volume is approximately 10 µL. Incubate the mixtures at room temperature for 5 min (Figure 3, see Note 8).
2. SYBR Green I: Add concentrated SYBR Green I to the DNA origami solution (10 nM) in a trisaminomethane-based buffer (see Note 9, 11) for desired final concentrations of SYBR Green I. Incubate the mixtures at room temperature for 2 h. Since SYBR Green I and EtBr share similar chemical structure, this protocol can also apply to EtBr. That is, EtBr may be used in place of SYBR Green I in this procedure (see Note 8).
3. BMEPC: Dilute the DNA origami to 1.0 nM in DI water containing BMEPC to reach different final concentrations from 5 to 25 µM of BMEPC. Incubate the mixtures at room temperature in dark condition for 12 h (see Note 8).
4. Daunorubicin: Add concentrated (250 µM) daunorubicin to 10 nM DNA origami solution in a trisaminomethane-based buffer (see Note 10, 11) to have various final concentrations of daunorubicin. Incubate the mixtures for different duration of time (all the way up to 24 h, see Note 8, 12).

### 3.6. UV Irradiation

1. Set the UVP lamp to “short-wavelength” (254 nm, see Note 13).
2. Dilute DNA origami ribbons (see Note 14) to 2 nM 20–40 µL using MES buffer and keep the solution in quartz tubes. Place the tubes at about 1 cm in front of the UVP lamp (or the UV Transilluminator).
3. Turn the UVP lamp (or the UV Transilluminator) on for less than 5 min (see Note 15) (Figure 4).

### 3.7. UVA Irradiation with Photoactive Chemical Adducts

1. Set the UVP lamp to “long-wavelength” (366 nm, see Note 16).
2. Dilute DNA origami ribbons to 2 nM 20–40 µL by MES buffer and saturated TP1 solution for a final volume ratio of TP1 solution to mixture at 1:10. Keep the mixture in quartz tubes and place the tubes at about 1 cm in front of UVP lamp.
3. Turn the UVP lamp on for less than 20 min (Figure 5).

### 3.8. Sample Preparation for AFM Imaging

All DNA origami samples are deposited onto mica surface before they are scanned under AFM.

1. Deposition of origami cylinders and tiles: Dilute origami cylinder, tiles, or cyclized tiles to 0.5 nM with TAEM buffer. Pipette 10 µL aliquot onto freshly cleaved mica surface and incubate for about 5 min at room temperature. After that, use compressed air to blow the mica dry and rinse with 80 µL DI water for about 3 s. At last, blow the mica dry again to keep it from contamination.
2. Deposition of elongated origami tubes and polymerized ribbons: Dilute elongated tubes or polymerized ribbons to 2 nM with MES buffer (see Note 17). Pipette 10 µL aliquot onto freshly cleaved mica surface and incubate for about 5 min at room temperature. Then, add 20 µL fixing buffer (see Note 17) to the same mica surface and incubate for another 2 min at room temperature. After that, use compressed air to blow the mica dry and rinse with 80 µL DI water for about 3 s. Finally, blow the mica dry again and keep it from contamination.

### 3.9. AFM Imaging

Use the Peak-Force tapping mode in a Bruker Dimension Icon AFM with SCANASYST-AIR probes to scan the surface of mica with DNA origami sample deposited on it. Perform multiple scans by selecting different places on the mica until a clear vision is acquired.

## 4. Notes

1. The unit of a DNA cage uses a slightly different 8064-nt scaffold derived from M13. There are a number of similar scaffold strands available from different manufacturers. The choice should be made by the need of length, the application (e.g., cleavage sites for restriction enzymes), and the cost. The scaffold concentration is approximately 50 nM, and staples are six times higher. Since the cage is distinct and different from the origami tile or cylinder, its conditions are also different. The key point here is that staples should always be excessive.
2. The unit of a DNA cage uses another trisaminomethane-based buffer (termed Tris-2 buffer) for annealing. It contains 5 mM trisaminomethane-Hydrochloric acid, 1 mM EDTA, and 12.5 mM magnesium chloride (pH 8.0). It is important that the pH and magnesium concentration are the same as those in TAEM buffer. These two conditions will determine whether the DNA origami will form or not. To achieve a better quality, detailed concentrations may be adjusted.
3. Staples usually come in by 96-well plates. In each well, the concentration is 75 µM. After mixing all the staples for DNA origami together, the concentration of each staple is 75 µM divided by the number of staples. For example, if there are 75 staples, then each staple is 1 µM; if 150 staples, then 0.5 µM. Therefore, depending on the total number of staples, the concentration of each staple in the mixture is different. To reach 3.5x staple concentration (relative to the scaffold), the amount of staple mixture needed will be different. Thus, the final volume is not going to be exactly 55 µL. If one wants to make more origami structures, the volume of each components will be greater and the total volume will increase accordingly.
4. The annealing temperature and duration for DNA nanocages range from 80 to 24 °C in 15–72 h. The exact annealing time depends on the target structure [64]. One example is holding at 80 °C for 5 min, decreasing to 65 °C at 5 min/°C, incubating at 65 °C for 20 min, and decreasing to 25 °C at 20 min/°C (for a total of 15 h) [65]. To repeat the work, one can have two options: 15- or 72-h annealing. To generate the protocol for 72-h annealing, the durations can be adjusted by the ratio of 15:72. For example, the first step should begin at 80 °C for 24 min.
5. The number of linkers (or releasers) is typically 10 to 20 in a DNA origami structure, while more than 100 staples are used. The concentration of the linker (releaser) mixture is 10-folds higher than that of the staples. For a desired concentration, approximately 2 µL of linker/releaser mixture is needed. When there is a linking and releasing cycle, the total volume will be higher than 57 µL.
6. In theory, the linkers can connect unlimited numbers of origami together. It is thus difficult to know the concentration of assembled or polymerized origami. For simplicity, their concentration is noted as that of the origami units, unless specified otherwise.
7. The conditions for reconfiguring DNA nanocages are different from those for origami cylinders. This can be understood as follows. The DNA reactions for reconfiguration are toehold-mediated strand displacement and reannealing. These two take place in seconds or minutes when the strands are in proximity [61]. However, the reacting strands have to diffuse to the reaction sites for a DNA origami structure, which also depends on the concentrations. To ensure the complete reactions, time and temperature are adjusted based on origami structures. Therefore, the conditions for two structures may be different.
8. EtBr and SYBR Green I have similar chemical structures. As such, their conditions are similar. If the origami concentration is 10 nM instead of 2 nM, incubation time increases from 5 min to 2 h to ensure that all the structures are intercalated. BMEPC and daunorubicin have more complex structures, thus it might take half to a full day for insertion into the base-pairs. Depending on the intercalator, other conditions (buffer, temperature, etc.,) differ slightly. For a safe start, one can use TAEM buffer with 2 nM DNA origami. By adding the intercalator of interest at various concentration up to few µM and incubating at room temperature for a day, the results can confirm the applicability of the intercalator.
9. The trisaminomethane-based buffer used for SYBR Green I (termed Tris-3 buffer) contains 40 mM trisaminomethane, 20 mM acetic acid, 1 mM EDTA disodium salt, and 40 mM magnesium chloride (pH 7.5).
10. Daunorubicin uses a trisaminomethane-based buffer containing 5 mM trisaminomethane, 5 mM sodium chloride, 1 mM EDTA, and 20 mM magnesium chloride (pH 8, termed Tris-4 buffer).
11. From the comparison of TAEM and Tris-2 to -4 buffers, trisaminomethane based buffers may have various concentrations and chemical additives (also see Note 2, 9 and 10). For example, the concentration of the buffer agent trisaminomethane can vary from 5 to 40 mM. In addition, some buffer contains sodium chloride, while others do not have chloride ions. In general, the buffers maintain a stable pH value and compensate for the charge interaction between DNA backbones with magnesium ions.
12. There are two additional methods to intercalate daunorubicin. Additional method 1: Mix 5–240 nM DNA origami solution in Tris-4 buffer (see Note 10) with 500 µM daunorubicin and incubate for 24 h. Additional method 2: Mix 62.5–2500 µM daunorubicin with 20 nM DNA origami solution in Tris-4 buffer (see Note 10) for 24 h. Among the three methods (one in the Methods and two in the Notes), the maximum incubation time is the same. This implies that daunorubicin needs about a day to fully intercalate into DNA structures.
13. UVC has short wavelengths (100 to 280 nm), corresponding to high-energy photons (4.4 to 12.4 eV). UVB has medium wavelengths (280 to 315 nm) or mid-energy photons (3.9 to 4.4 eV). Both can relax the stress of DNA origami directly.
14. One may irradiate UV light on DNA origami monomers and then polymerize them into ribbons. This may create minor photolesions in the single-stranded domain of the scaffold, which may affect the hybridization of the origami with the linkers for polymerization (see Note 15). Therefore, the polymerization in the second step may not work properly.
15. In order to not damage DNA origami, UV dose should be restricted. The limits are approximately 8.3 and 20.3 kJ/m^2^ for UVC and UVB, respectively. For the best flattening result, the dose should be around 2.5 and 6.8 kJ/m^2^ for UVC and UVB, respectively.
16. The UVA wavelength ranges from 315 to 400 nm, corresponding to low energies (3.1 to 3.9 eV). UVA light does not relax the stress in DNA origami directly. Instead, UVA may be used to control photoactive chemical adducts (such as TP1/TP2), thereby modulating the stress and conformation of DNA origami.
17. For deposition processes, especially when the sample is elongated tubes or ribbons, appropriate adhesion and firm fixing on mica surface are needed. This is crucial for origami ribbons so that near uniform gaps between the kinks can be imaged clearly (e.g., Figure 3f, AFM image). Both MES buffer and fixing buffer should be used.

## 5. Concluding Remarks

In this work, we show reliable, robust methods and protocols to transform the DNA configurations by using DNA oligomers, chemical adducts, and external UV light. Related shape-changing mechanisms are articulated. This protocol paper provides additional details and discussions of the published works, particularly for new graduate students and the researchers from other fields who wish to use DNA origami in their application areas. We envision that this will help researchers adapt the methods.

The described protocols can control the origami configurations in a progressive, controllable manner for engineering applications. These methods may be exploited to use DNA nanostructures as actuators or dynamic templates for other molecules. The DNA origami nanocages discussed above is a good example for using DNA oligomers to control the DNA configurations that serve as a template to modulate liposomes.

While the DNA origami-caged liposomes are beautifully demonstrated, the reconfiguration has some limitations on the number of possible states. There are three distinct states: long (closed), short (open), and bended (partial open) states. This could be improved by implementing a progressive control. One could imagine that with DNA intercalators, the proximity of individual liposomes in the nanocages could be adjusted gradually. Therefore, it may allow for studying the merging process in many aspects. For instance, the threshold for vesicle fusion and the stability of a merged single liposome as a function of the distance. In addition, the nanocages may be bended progressively, and thus, liposomes with different curvatures can be compared. By inspecting the differences between the DNA template and the liposome, a preferred curvature range could be found.

Another potential application could be developed on a previous work by Qian and coworkers [66]. They successfully demonstrated a fractal assembly of different patterns at micron scale, including Mona Lisa’s face, rooster, and other 2D geometries. One possible enhancement could be made to control their shapes in response to UVA light with photoswitchable intercalators. Once the molecules are switched into intercalators by UVA, the shape of the pattern may change drastically. The changes could be visualized under an optical microscope, which is faster and easier than AFM or TEM. The system might be developed for an environmental UV radiation sensor.

Finally, it is also possible to build nanoscale actuators using dynamic DNA nanostructures. DNA shafts or rods are commonly constructed by many research groups [42,67,68]. The rods, when made stiff, would have a fixed number of helical turns from one terminal to the other. By introducing UV light (UVB or UVC), the helicity of DNA will be changed, and thus, the turns in the rods will be altered in a gradual fashion. Ultimately, the rods might act as an irreversible stepper motor, an essential component in nanoscale machines.

## Data Availability

Not applicable.
